# Supplementary material for: The interactome of intact mitochondria by cross-linking mass spectrometry provides evidence for coexisting respiratory supercomplexes
Source: Mol Cell Proteomics. 2017 Dec 8;17(2):216–32. doi: 10.1074/mcp.RA117.000470 (PMC5795388; doi:10.1074/mcp.RA117.000470)
Supplement: Supplemental Data [file supp_RA117.000470_133922_0_supp_23978_fzffwf.zip › spectra_annotation/mito_spectra_annotation/231-1-1-1-10-1.pdf]

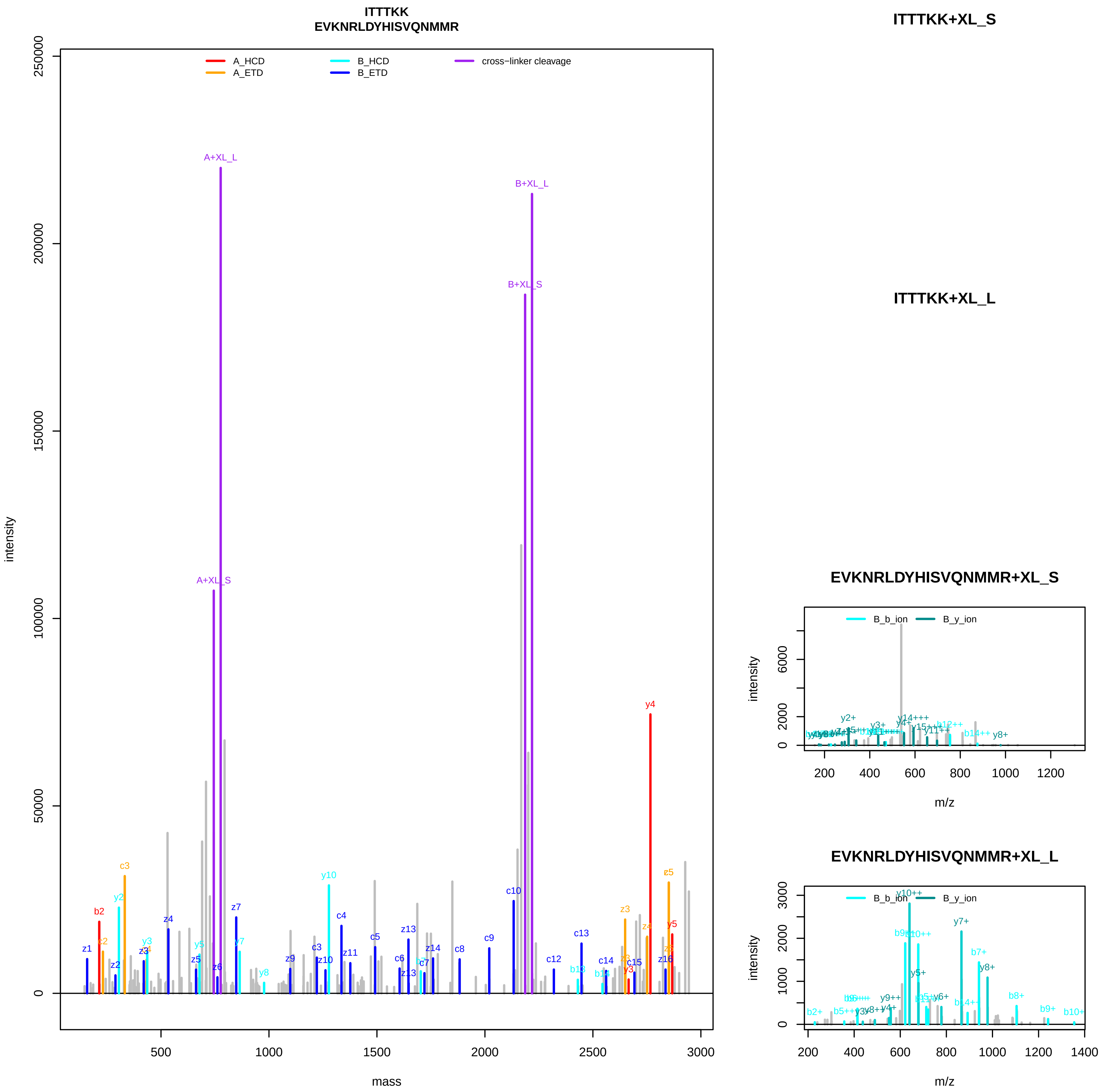

ITTTKK
EVKNRLDYHISVQNMMR
A_HCD
A_ETD
B_HCD
B_ETD
cross−linker cleavage
intensity
mass
0
50000
100000
150000
200000
250000
500
1000
1500
2000
2500
3000
A+XL_L
A+XL_S
B+XL_L
B+XL_S
y4
c3
y10
c10
c5
z1
b2
c2
y2
z2
z3
y3
z4
z5
y5
z6
z7
y7
y8
z9
c3
z10
c4
z11
c5
c6
z13
c7
z14
c8
c9
c12
c13
c14
c15
z16
y5
z3
y3
z4
ITTTKK+XL_S
ITTTKK+XL_L
EVKNRLDYHISVQNMMR+XL_S
B_b_ion
B_y_ion
intensity
m/z
0
2000
6000
200
400
600
800
1000
1200
y2+
y14+++
y4+
y3+
b12++
y15++
y11++
b14++
y8+
EVKNRLDYHISVQNMMR+XL_L
B_b_ion
B_y_ion
intensity
m/z
0
1000
2000
3000
200
400
600
800
1000
1200
1400
y10++
b9++
b10++
y7+
b7+
y8+
y5+
b8+
b2+
b5++
y9++
y4+
y6+
b14++
b9+
b10+
